# Networked T Cell Death following Macrophage Infection by *Mycobacterium tuberculosis*

**DOI:** 10.1371/journal.pone.0038488

**Published:** 2012-06-04

**Authors:** Stephen H.-F. Macdonald, Elliott Woodward, Michelle M. Coleman, Emma R. Dorris, Parthiban Nadarajan, Wui-Mei Chew, Anne-Marie McLaughlin, Joseph Keane

**Affiliations:** Department of Clinical Medicine, Trinity Institute of Molecular Medicine, St. James’s Hospital, Dublin, Ireland; Fundació Institut d’Investigació en Ciències de la Salut Germans Trias i Pujol. Universitat Autònoma de Barcelona. Ciberes, Spain

## Abstract

**Background:**

Depletion of T cells following infection by *Mycobacterium tuberculosis* (Mtb) impairs disease resolution, and interferes with clinical test performance that relies on cell-mediated immunity. A number of mechanisms contribute to this T cell suppression, such as activation-induced death and trafficking of T cells out of the peripheral circulation and into the diseased lungs. The extent to which Mtb infection of human macrophages affects T cell viability however, is not well characterised.

**Methodology/Principal Findings:**

We found that lymphopenia (<1.5×10^9^ cells/l) was prevalent among culture-positive tuberculosis patients, and lymphocyte counts significantly improved post-therapy. We previously reported that Mtb-infected human macrophages resulted in death of infected and uninfected bystander macrophages. In the current study, we sought to examine the influence of infected human alveolar macrophages on T cells. We infected primary human alveolar macrophages (the primary host cell for Mtb) or PMA-differentiated THP-1 cells with Mtb H37Ra, then prepared cell-free supernatants. The supernatants of Mtb-infected macrophages caused dose-dependent, caspase-dependent, T cell apoptosis. This toxic effect of infected macrophage secreted factors did not require TNF-α or Fas. The supernatant cytotoxic signal(s) were heat-labile and greater than 50 kDa in molecular size. Although ESAT-6 was toxic to T cells, other Mtb-secreted factors tested did not influence T cell viability; nor did macrophage-free Mtb bacilli or broth from Mtb cultures. Furthermore, supernatants from *Mycobacterium bovis* Bacille de Calmette et Guerin (BCG)- infected macrophages also elicited T cell death suggesting that ESAT-6 itself, although cytotoxic, was not the principal mediator of T cell death in our system.

**Conclusions:**

Mtb-Infected macrophages secrete heat-labile factors that are toxic to T cells, and may contribute to the immunosuppression seen in tuberculosis as well as interfere with microbial eradication in the granuloma.

## Introduction

Tuberculosis (TB) in an immunosuppressive illness and lymphopenia often occurs in TB patients [1], [2]. Rather than being an epiphenomenon, it is more likely that this T cell deficiency contributes to pathogen persistence in the host, and the lack of a meaningful immune response during chronic TB infection. Indeed, intracellular pathogens such as *Mycobacterium tuberculosis* (Mtb) must suppress immunity to survive within an infected host [3], [4], and the status of host T lymphocytes is a critical factor in determining the resolution of chronic infections, like tuberculosis. The mechanism of lymphopenia in tuberculosis patients is poorly understood, but may involve activation-induced apoptosis or sequestration of lymphocytes to inflamed organs such as the lung.

T cells enhance the activity of phagocytes against Mtb and other intracellular microbes by delivering activating signals, including interferon-γ (IFN-γ) [5], subsequently upregulating key processes such as nitric oxide generation and apoptosis [6], [7]. Accordingly, the significant T cell depletion which accompanies active TB disease is associated with poor prognosis [1], [8] as well as diminished cytokine responses which may persist even following successful antitubercular therapy [9]. T cell apoptosis is also an abundant feature of the granuloma, which is a highly organised structure comprising a necrotic centre containing bacteria, dead and infected macrophages, as well as multinucleate giant cells, surrounded by a peripheral cuff of lymphocytes [10], [11]. Host phagocyte death can be inhibited by Mtb [12], and it has been shown, by Mustafa *et al*., that infected cells at the granuloma centre express reduced levels of apoptotic markers such as caspase 3 than those that are uninfected [13]. However significant levels of *pro*apoptotic surface molecules such as Fas ligand are expressed at the periphery of the granuloma, where T cells accumulate [14]. This may represent an infection-induced ‘keep out’ signal that prevents T cell penetration into the granuloma centre. Additionally, Hirsch *et al*. have described an increased susceptibility to apoptosis in T cells taken from tuberculosis patients [15], and research by Sharma *et al*. indicated that infected macrophages can signal T cell apoptosis [16]. The mechanism of this T cell death response is unclear, and it is not known if Mtb-infected human alveolar macrophages (AMs) can supply this immunosuppressive death signal.

We found that lymphopenia was prevalent in our culture positive tuberculosis patients, and there was a statistically significant recovery of lymphocyte counts after anti-TB treatment. Because animal model work had associated low lymphocyte counts with death following mycobacterial infection [17], we sought to investigate the toxic effect of infected macrophages on T cells. We have previously reported that human alveolar macrophage infection by Mtb can elicit signals that kill infected cells and nearby uninfected bystander macrophages, in what is likely a host-protective response. The macrophage death response deprives the bacilli of their replicative niche cell and facilitates delivery of mycobacterial antigens to APCs, enabling antigen presentation [18], [19]. Even as the infected human alveolar macrophage death response may support TB immunity, it is likely that a death signal delivered to nearby T cells would diminish the host response [12], [20]. We now report that Mtb-infected human alveolar macrophages do cause networked, caspase-dependent lymphocyte apoptosis. Like alveolar macrophages, cell-free culture supernatants from Mtb-infected THP-1 macrophages reliably induced this T cell killing phenotype. We found that this human macrophage cell line recapitulates this host response to infection, which is dose-dependent, but TNF-α and Fas-independent. We show that macrophage-free Mtb bacilli are not sufficient to signal T cell death, which suggests a role for a PAMP/DAMP complex derived from infected cells. Thus far, we have begun to characterise the T cell death-inducing signal secreted from infected macrophages, and found it to be heat-labile and greater than 50 kDa in molecular size.

**Figure 1:**
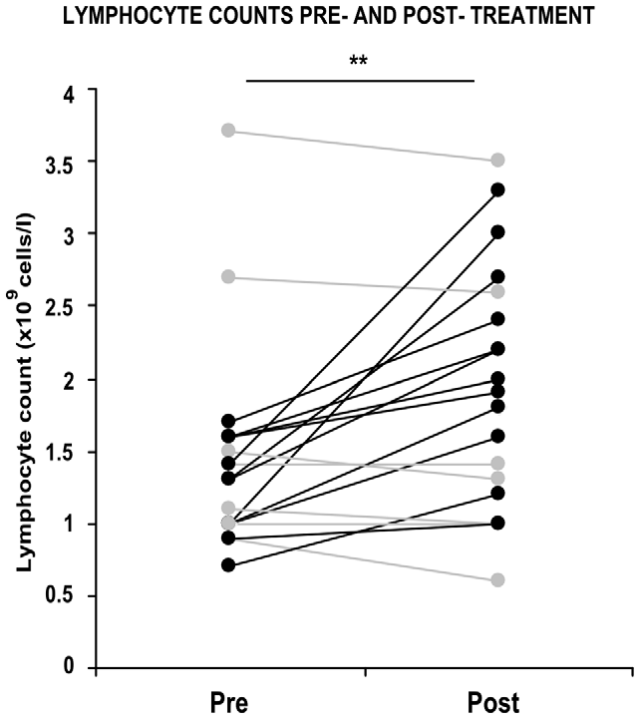
Patient lymphocyte counts pre- and post antitubercular chemotherapy. Lymphocyte counts were taken, before and after treatment, from twenty patients who were culture-positive for Mtb. Each joined pair of data points represents pre- and post-therapy lymphocyte counts (×10^9^ cells/l) from a single patient. Data from patients whose lymphocyte counts recovered following treatment are shown in black; counts from patients whose lymphocyte counts fell or did not change are shown in grey. ** =  p<0.01, paired Student’s t test comparing pre-treatment vs post-treatment counts.

## Methods

### Ethics Statement

For patient lymphocyte counts, all analysis was performed anonymously on preexisting clinical data, therefore consent was not required. For alveolar macrophage isolation, informed written consent was obtained as part of the project “Airway gene expression profiling and immune response to lung disease”, approved by the St. James’s Hospital/AMNCH Research Ethics Committee. Only the “Immune Response to Lung Disease” aspect of the ethics approval was used in this manuscript.

### Reagents and Antibodies

Except where specified, all reagents and materials were obtained from Sigma-Aldrich. Anti-human TNF-α blocking antibody (cat# MAB210) and mouse IgG1 isotype control (cat# MAB002) were obtained from R&D systems. Anti-human Fas blocking antibody ZB-4 (cat# 05-338) was obtained from Millipore. Mtb secreted factors 16 kDa (cat# NR-14860), 45 kDa (cat# NR-14862), Antigen85 (cat# NR-14855), ESAT-6 (cat# NR-14868), GroES (cat# NR-14861), ManLAM (cat# NR-14848) and Phos1 (cat# NR-14859) were obtained from BEI resources, NIAID, NIH under the TB Vaccine Testing and Research Materials (TBVTRM) contract no. HHSN266200400091C awarded to Colorado State University.

### Patient Lymphocyte Counts

All analysis of patient data was carried out anonymously. We performed a retrospective analysis of lymphocyte counts of twenty Mtb-culture positive patients recruited consecutively from our TB service at St James’ Hospital, starting November 2009. Pre-treatment lymphocyte counts were performed on the date of diagnosis prior to initiating anti-tuberculous treatment and post treatment lymphocyte counts were performed after at least 2 months of treatment. Lymphopenia was defined as a lymphocyte count of less than 1.5×10^9^/l. Immunosupressed patients (e.g. HIV-positive, steroid use) and cancer patients were excluded.

**Figure 2:**
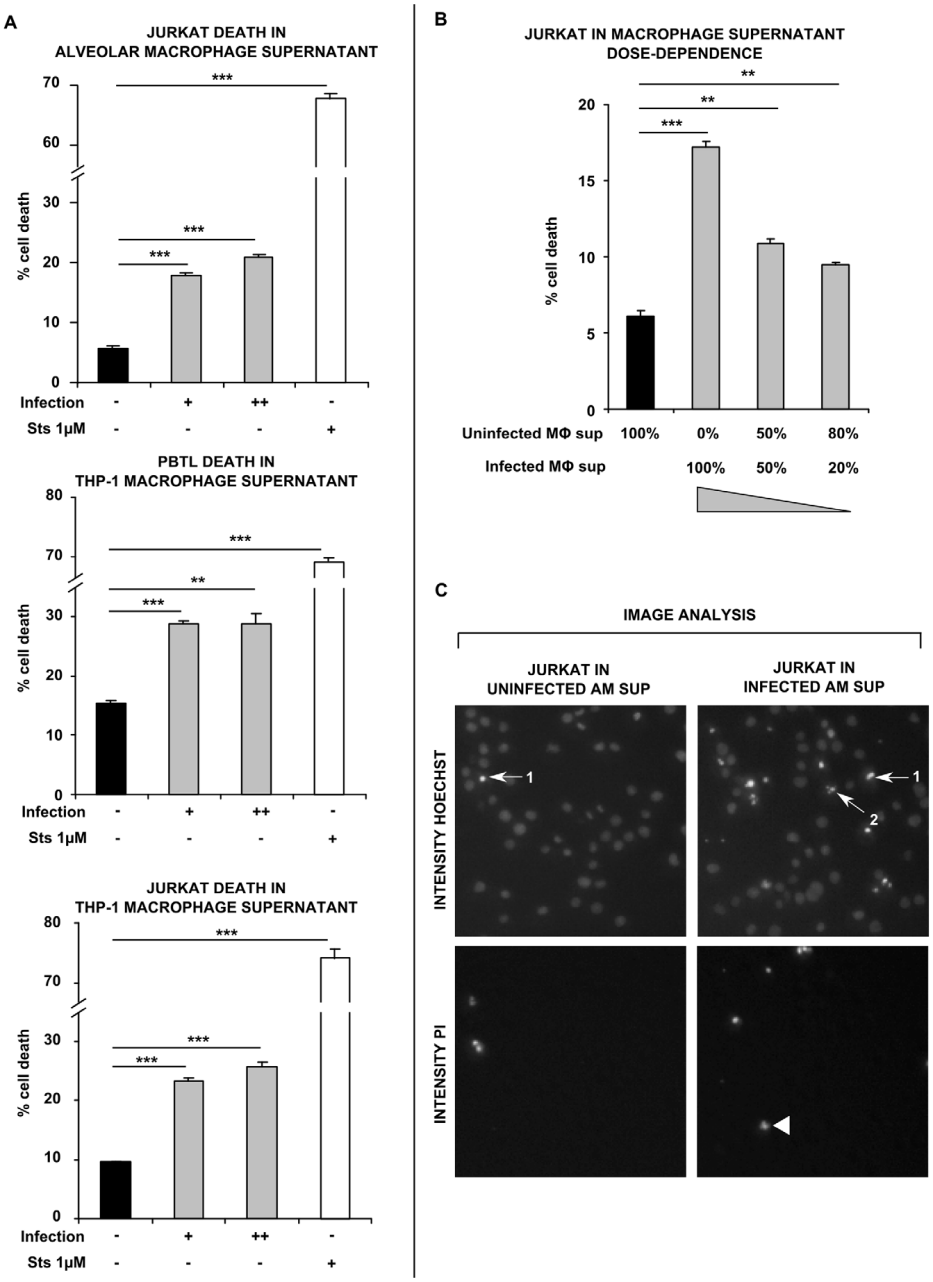
*M.tuberculosis*-infected macrophage supernatants are toxic to T cells. (A) Jurkat T cells underwent significantly increased cell death in cell-free supernatants of low MOI and high MOI Mtb-infected human primary alveolar macrophages (AMs) (shaded bars; infection + and ++, respectively), compared to those of uninfected AMs (closed bars, infection –). Staurosporine 1 µM was used as a positive control to induce cell death (open bars). Similar effects were observed on PBTL and Jurkat T cells exposed to the cell-free supernatants of uninfected/infected THP-1 macrophages. (B) Jurkat cell death in infected THP-1 macrophage supernatant occurred in a dose-dependent manner; infected macrophage supernatants were diluted to 50% and 20% in uninfected supernatant, reducing the level of T cell killing. (C) Example cropped fluorescence intensity images for Hoechst and PI staining from Jurkats incubated in uninfected and infected AM supernatants. Images were processed using automated INCell image analysis to detect apoptotic condensed and fragmented nuclei (arrow 1 and 2, respectively) and PI-positive cells (arrow head). Data in each panel are from one representative experiment, showing mean percentage cell death ± SEM from incubations performed in triplicate. ** =  p<0.01, *** =  p<0.001, Student’s *t* test relative to uninfected control.

### Culture of *Mycobacterium Tuberculosis*

*M.tuberculosis* H37Ra and *M.bovis* BCG, obtained from ATCC, were grown to log phase at 37°C in 5% CO_2_ in Middlebrook 7H9 broth (Difco), supplemented with albumin-dextrose-catalase (Becton Dickinson) and 0.05% Tween-80 (Difco), and made up in endotoxin-free water.

### Isolation of Primary Human Alveolar Macrophages

Human alveolar macrophages were obtained at bronchoscopy, after written consent, under a protocol approved by the St. James’s Hospital/AMNCH ethics board. Cells in bronchoalveolar lavage fluid were passed through a 100 µm nylon cell strainer (BD Bioscience), then centrifuged for 15×min at 200×g and subsequently resuspended in RPMI supplemented with 10% human serum, 0.2% Fungizone, and 0.1% Cefotaxime. AMs were then seeded onto tissue culture plates at a density of 5×10^5^ cells per ml, and incubated overnight at 37°C in 5% CO_2_ before use.

### Culture of Human Cells

Cells of the human T cell leukaemia line, Jurkat (ATCC #TIB-152), and the monocytic leukaemia line, THP-1 (ATCC #TIB-202) were routinely cultured at 37°C in 5% CO_2_ in RPMI 1640, supplemented with 10% FBS (hereafter referred to as ‘complete RPMI’), at a density of between 1×10^5^−1×10^6^ and 2×10^5^−8×10^5^ cells/ml respectively.

### Isolation of Primary Human Peripheral Blood T lymphocytes (PBTL)

Lymphocytes were isolated from human whole blood provided by the Irish Blood Transfusion Service. Briefly, blood was diluted 1∶2 with sterile PBS, then layered onto lymphoprep and spun at 1200×g without brake for 20 min at room temperature. Buffy coats were removed, and then washed three times with PBS. Cells were then resuspended in serum-free RPMI, and incubated for 2 h at 37°C in 5% CO_2_ to remove monocytes. Nonadherent cells were then harvested and resuspended in complete RPMI +2 µg/ml PHA and incubated for 3 days at 37°C in 5% CO_2_. Subsequently, cells were washed in complete RPMI and resuspended at a density of 1.5×10^6^ cells/ml in complete RPMI supplemented with 20 ng/ml IL-2 (Peprotech), then incubated for 5 days at 37°C in 5% CO_2_ to generate PBTLs.

### *M. tuberculosis* Infection of Macrophages

*M.tuberculosis* H37Ra or *M.bovis* BCG bacilli growing in the log phase were centrifuged at 3000×g for 10 min, and then resuspended in complete RPMI. This suspension was then passed 8 times through a 25 G needle, before centrifugation at 100×g for 3 min to remove remaining clumped bacteria. Bacterial suspension was then transferred to a fresh tube, and subsequently used for infection of AMs prepared as described above, or THP-1 cells, previously differentiated to a macrophage phenotype by 72 h incubation with 100 nM Phorbol 12-myristate 13-acetate (PMA). Cells were infected with a range of volumes of Mtb H37Ra or BCG suspension, then following 3 h incubation, remaining extracellular bacilli were washed off with PBS, and cells were fixed with 2% paraformaldehyde in PBS for 5 min, and subsequently stained using a Rapid Modified Auramine O Stain Set (Scientific Device Laboratory Inc.) according to the manufacturer’s protocol, followed by 5 min staining with 10 µg/ml Hoechst 33258. Cells were imaged using fluorescent microscopy in order to determine the Multiplicity of Infection (MOI), where a low MOI was defined as >70% of cells infected with 1–5 bacteria, and a high MOI as >80% of cells infected with 6–10 bacteria. Subsequently, AMs at a density of 5×10^5^ cells/ml or THP-1 macrophages growing at a density of 5×10^4^ cells/ml in tissue culture plates, were infected in this manner, incubated for 3 h, then remaining extracellular bacilli were washed off and infected cells were incubated for a further 48 h in complete RPMI. For uninfected controls, cells were processed in parallel, but without addition of bacilli. Following this incubation, supernatants were removed from the cells, then centrifuged at 6,800×g to remove any free bacteria, and stored at −20°C.

**Figure 3:**
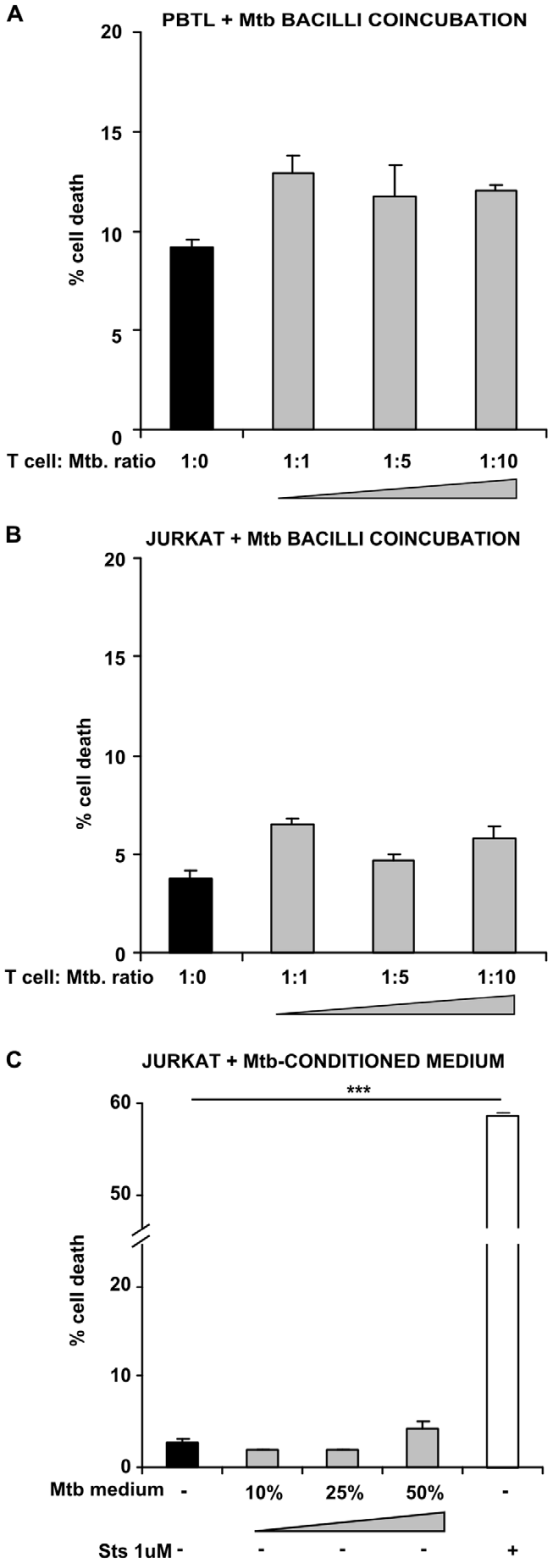
Macrophage-free *M.tuberculosis* bacilli are not sufficient to induce T cell death. PBTLs (A) and Jurkat T cells (B) were incubated either in complete RPMI only (closed bars) or coincubated with Mtb bacilli at a range of T cell: Mtb ratios (shaded bars). Subsequently, Jurkat T cells were incubated in complete RPMI supplemented with medium taken from Mtb growing in the log phase at a range of ratios (shaded bars) (C). Staurosporine 1 µM was used as a positive control to induce cell death. Data in each panel are from one representative experiment, showing mean percentage cell death ± SEM from incubations performed in triplicate. *** =  p<0.001, Student’s *t* test relative to uninfected control.

### T Cell Culture in Cell-free Supernatants from Mtb-infected Macrophages

Jurkat T cells or PBTLs were incubated in 96-well plates at a density of 1×10^4^ cells/well, in triplicate, in a volume of 150 µl/well, in cell-free supernatants from Mtb- or BCG- infected macrophages. 1 µM Staurosporine was used as a positive control for cell death. Following 24 h incubation, cells were stained simultaneously with 5 µg/ml Propidium Iodide (PI), 20 µg/ml Hoechst 33342 and 50 µg/ml Hoechst 33258, briefly resuspended by gentle pipetting, then plates were centrifuged at 400×g for 2 min to bring cells in suspension to the bottom surface of the well. Plates were then imaged using an INCell Analyzer 1000 cellular imaging system (GE Healthcare) and images were analyzed using GE INCell Analyzer 1000 Workstation software version 3.6 (GE Healthcare) as we have previously published [21]. Total cell numbers were detected via Hoechst staining of nuclei, and the dying/dead cells were identified via positivity for PI staining and/or nuclear condensation, which was characterised by elevated intensity of Hoechst staining within the nuclei (see figure S1). 12–18 fields of view per well were acquired.

### *M. tuberculosis*/T Cell Coculture, Supernatant and Secreted Antigen Experiments

For coincubation experiments, Mtb H37Ra bacilli growing in the log phase were incubated in 96-well plates with T cells at varying ratios, with a constant 1×10^4^ cells per well in triplicate, then imaged with Hoechst/PI staining as before. T cells were also incubated in triplicate in 96-well plates at 1×10^4^ cells per well for 24 h in increasing dilutions of cleared culture supernatant taken from Mtb growing in the log phase in free culture, and then imaged. For secreted antigen experiments, T cells were incubated in the same manner, in logarithmic dilutions of each antigen in a concentration range of 10 µg/ml to 0.1 µg/ml, for 24 h before imaging.

### Manipulation of Infected Macrophage Supernatants

For blocking antibody experiments, anti-human TNF-α (hereafter referred to as anti-hTNF-α) (5 µg/ml), anti-human Fas (hereafter referred to as anti-Fas) (2.5 µg/ml) or isotype control IgG at the same concentration were added to supernatants, and incubations carried out as described above. In positive control experiments for TNF-mediated cell killing, cells were incubated for 15 min in the presence of 0.2 µg/ml cycloheximide, then recombinant human TNF-α (R&D Systems) was added to 5 ng/ml. In positive control experiments for Fas ligand-mediated cell killing, cells were incubated in the presence of 10 ng/ml recombinant human Fas ligand (R&D systems), crosslinked with 10 µg/ml anti-6× Histidine (R&D systems).

For heat treatment experiments, supernatants were heated to 95°C for 20 min, and then allowed to cool to room temperature before use. Initial experiments (data not shown) indicated that to preserve normal T cell viability, medium heated in this manner requires subsequent resupplementation with 10% FBS, therefore all supernatants were resupplemented following heat treatment.

For molecular weight cutoff experiments, supernatants were spun on Amicon Ultra-15 centrifugal filter units with Ultracel-50 membrane (Millipore), then filtrate (hereafter referred to as <50 kDa fraction) and retentate (hereafter referred to as >50 kDa fraction) recovered. During optimisation experiments (data not shown), the >50 kDa fractions were found to affect the basal levels of cell death when applied to T cells, due to concentration of soluble factors from the medium itself, therefore fractions were diluted to 25% or 10% in fresh medium for subsequent experiments.

### Statistical Analysis

In data from patients who underwent antitubercular chemotherapy, a two-tailed, paired Student’s *t* test was used in Microsoft Excel to determine significance between pre- and post-treatment lymphocyte counts. In all other cases, experiments were carried out in triplicate, and were performed a minimum of three times, except where specifically stated otherwise, with representative data from one experiment shown in each figure. Results were expressed as mean ± SEM, and statistical analysis was performed using a two-tailed, unpaired Student’s *t* test in Microsoft Excel. A *p*-value of less than 0.05 was considered to be statistically-significant.

**Figure 4:**
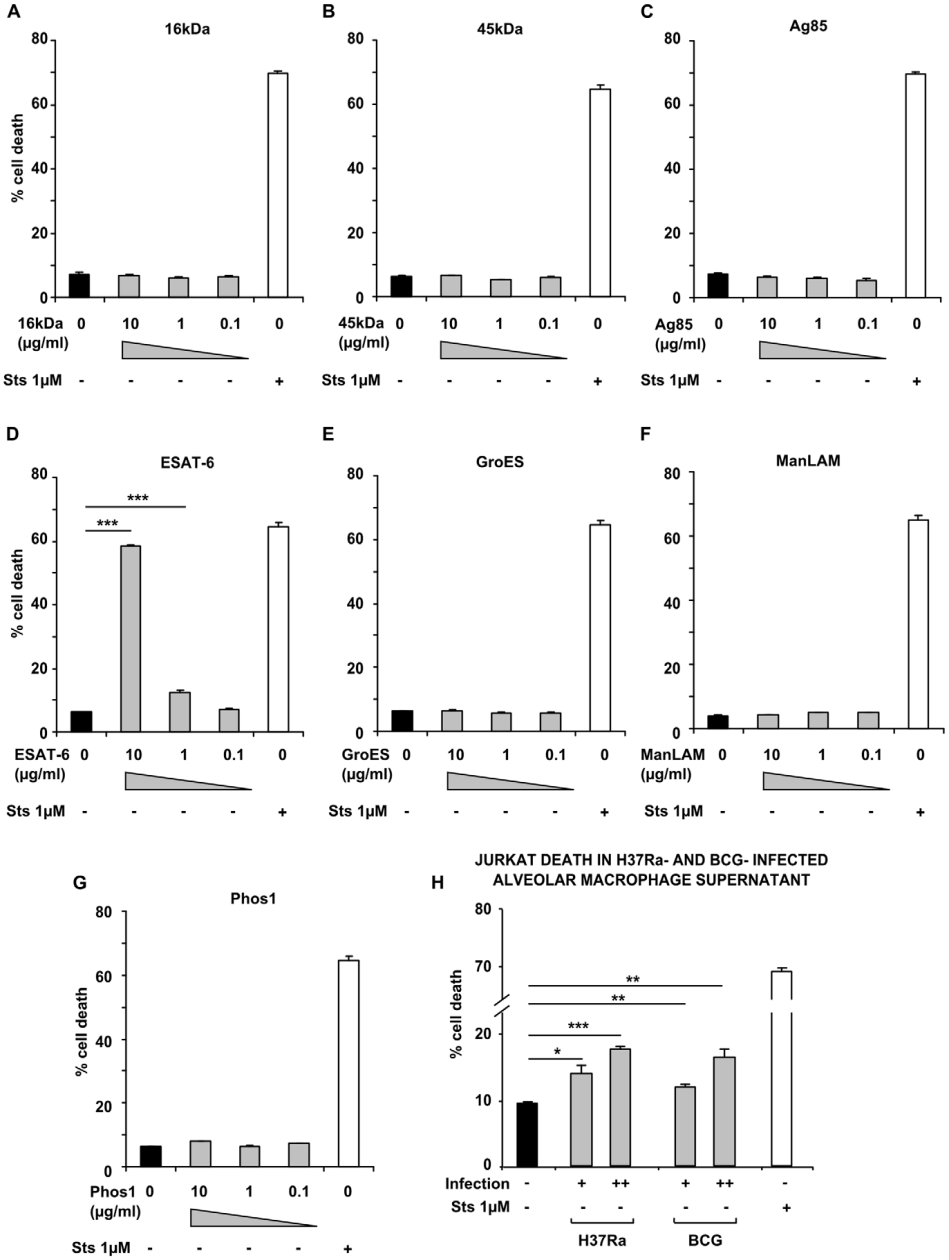
T cell death after exposure to *M.tuberculosis* secreted antigens. Jurkat T cells were exposed for 24 h to a panel of secreted factors derived from Mtb. No increase in cell death occurred when T cells were incubated with 16 kDa, 45 kDa, Antigen 85 complex, GroES, ManLAM or Phos1 (A–C, E–G, respectively, shaded bars), however elevated death occurred in cells incubated with ESAT-6 at 10 µg/ml or 1 µg/ml (D). 1 µM Staurosporine was used as a positive control in each case (open bars); vehicle control was either PBS or DMSO in the case of ManLAM (closed bars). Supernatants of AMs infected with H37Ra or *M.bovis* BCG both significantly increased T cell death (H, shaded bars), compared with uninfected AM supernatants (H, closed bars). Data in each panel are from one representative experiment, showing mean percentage cell death ± SEM from incubations performed in triplicate. * =  p<0.05, ** =  p<0.01, *** =  p<0.001, Student’s *t* test relative to vehicle (A–G) or uninfected (H) control.

## Results

### Lymphopenia is Prevalent in Tuberculosis Patients and Recovers after Treatment

We assessed the lymphocyte counts of twenty consecutively recruited patients who were culture-positive for Mtb infection, sampled before and after antitubercular chemotherapy. Low levels of lymphocytes (<1.5×10^9^/l in 13 out of 20 cases) were prevalent at presentation, but significantly improved after treatment (p<0.01) (fig. 1). This suggests that primary lymphopenia is not the reason for tuberculosis reactivation in this group. It does however link infection burden and inadequate lymphocytes [22]. We then investigated the signaling factors released from Mtb-infected human macrophages that contribute to this clinical phenotype, of lymphocyte death.

### *M. tuberculosis* Infection of Primary Human Alveolar Macrophages Signals T Cell Death

To investigate whether soluble mediators released during Mtb infection could alter the viability of T cells in a contact-independent manner, we first infected primary human alveolar macrophages for 48 h with Mtb H37Ra bacilli, then harvested culture supernatants and applied them to Jurkat T cells for 24 h before assessing cell death. In Jurkats incubated in cell-free supernatants from uninfected AMs, 5.85±0.13% cell death was observed, whereas in supernatants from low and high MOI Mtb-infected AMs, this increased significantly (p<0.001) to 18.12±0.24% and 21.13±0.25%, respectively. 1 µM Staurosporine used as a positive cell-killing control caused 67.33±0.85% death (fig. 2A). In this, and subsequent experiments, an automated image analysis strategy (figure S1) was used to clearly identify apoptotic cells that had condensed/fragmented nuclei and/or PI staining, and discriminate them from live cells (fig. 2C).

In the past, we have found that PMA-treated THP-1 human macrophages serve as a good model for the human alveolar macrophage, with respect to innate responses after infection with Mtb. Specifically we have shown that the host responses of infection-induced macrophage apoptosis and phagolysosomal maturation arrest are well reproduced in this cell-line [23], [24]. To test whether PMA-treated THP-1 cells could be used as a model human macrophage to study the mechanism of networked T cell death, we investigated the toxic effect of infected THP-1 macrophage supernatants on T cells. Peripheral blood T lymphocytes were incubated for 24 h in supernatants from Mtb-infected THP-1 macrophages, or supernatants from uninfected macrophages as controls. 15.33±0.60% cell death occurred in PBTLs exposed to uninfected supernatants, whilst in those incubated in low and high MOI supernatants, this increased significantly to 28.80±0.41% and 28.81±1.53% (p<0.001 and 0.01), respectively (fig. 2A). In order to investigate whether PBTLs could be replaced with a cell line in further studies, we repeated this experiment using the Jurkat T cell line as a death signal target. In Jurkat cells cultured in supernatants from uninfected THP-1 macrophages, 9.57±0.03% cell death was observed following incubation, whereas in cells cultured in low MOI and high MOI-infected macrophage supernatants, significantly (p<0.001) enhanced cell death was observed, with percentage dead cells increasing to 23.33±0.60% and 25.68±0.69% respectively (fig. 2A). Staurosporine was used routinely as a positive control, and significantly (p<0.001) increased cell death to 68.45±0.57% and 72.61±1.41% in PBTLs and Jurkats, respectively.

**Figure 5:**
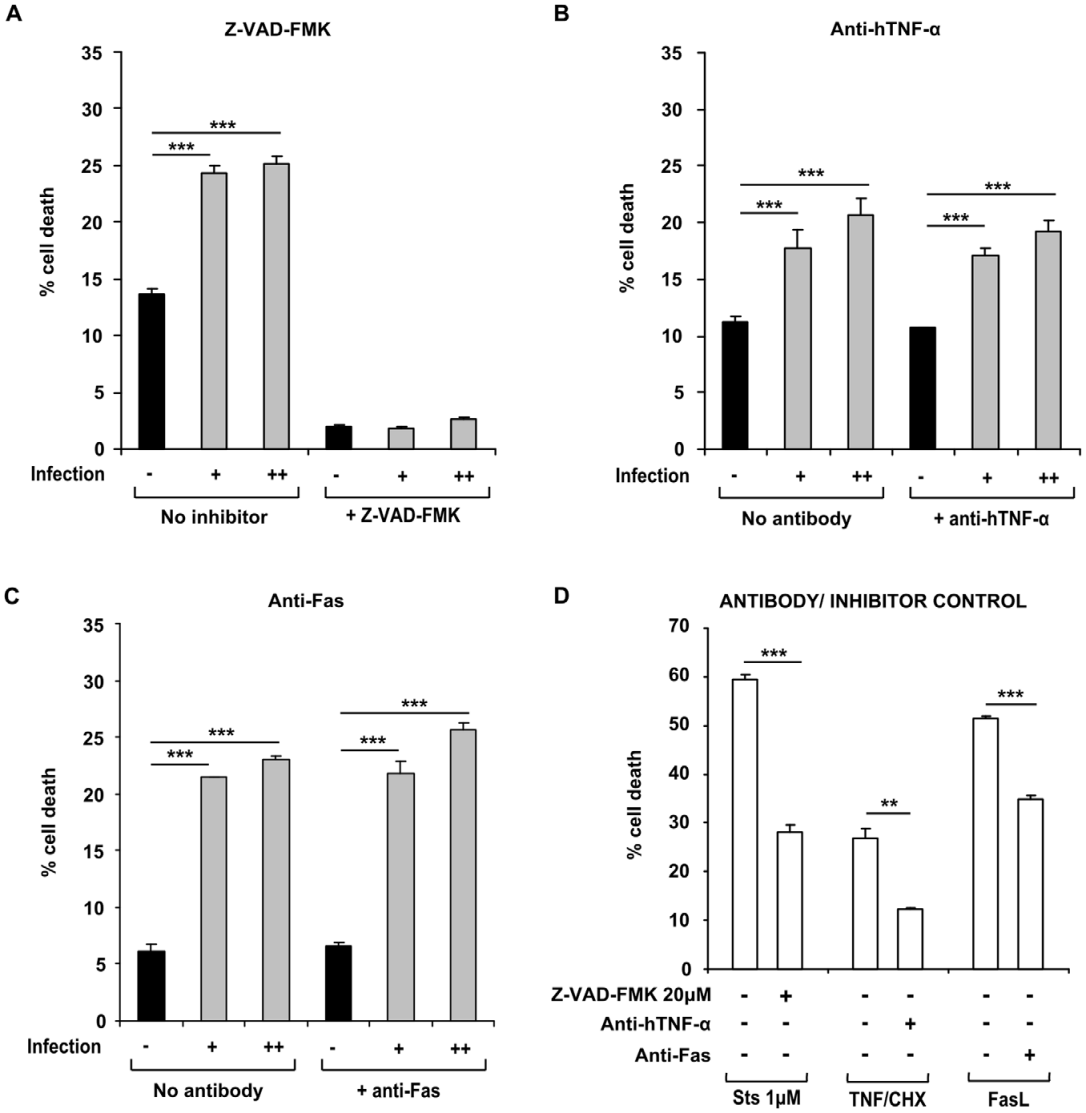
T cell death in *M.tuberculosis*-infected macrophage supernatants is caspase-dependent, and independent of TNF-α and Fas. (A) Addition of the pan-caspase inhibitor Z-VAD-FMK mitigated cell death induced by low MOI and high MOI-infected macrophage supernatants (shaded bars, infection + and ++ respectively), as well as background cell death observed in the uninfected control supernatants (closed bars, infection -). Cell death in infected supernatants was not mitigated by coincubation with blocking antibodies to TNF-α (B) or Fas (C), and in each case was significantly higher than in uninfected control supernatants (closed bars). Cell death induced by positive controls (D) (1 µM Staurosporine, 5 ng/ml recombinant human TNF-α +0.2 µg/ml cycloheximide and 10 ng/ml recombinant human Fas ligand) was in each case significantly reduced by coincubation with the corresponding inhibitor (open bars). Data shown in each panel are from one representative experiment, showing mean percentage cell death ± SEM from triplicate incubations. ** =  p<0.01, *** =  p<0.001, Student’s *t* test relative to uninfected control (A–C), or positive control relative to inhibitor (D).

### T Cell Killing by Infected Macrophage Supernatants is Dose-dependent

To confirm that soluble mediators contained in the medium of infected macrophages were responsible for T cell death, we carried out a dose-response assay using infected supernatants. In uninfected control THP-1 supernatants, 6.11±0.36% Jurkat T cell death was observed. This was significantly (p<0.001) elevated in undiluted, high MOI-infected THP-1 supernatant, to 17.23±0.49%, and progressively reduced to 10.88±0.45% at a 50% dilution, and 9.49±0.42% at a 20% dilution, both of which were also significantly (p<0.01) higher than the uninfected control (fig. 2B).

**Figure 6:**
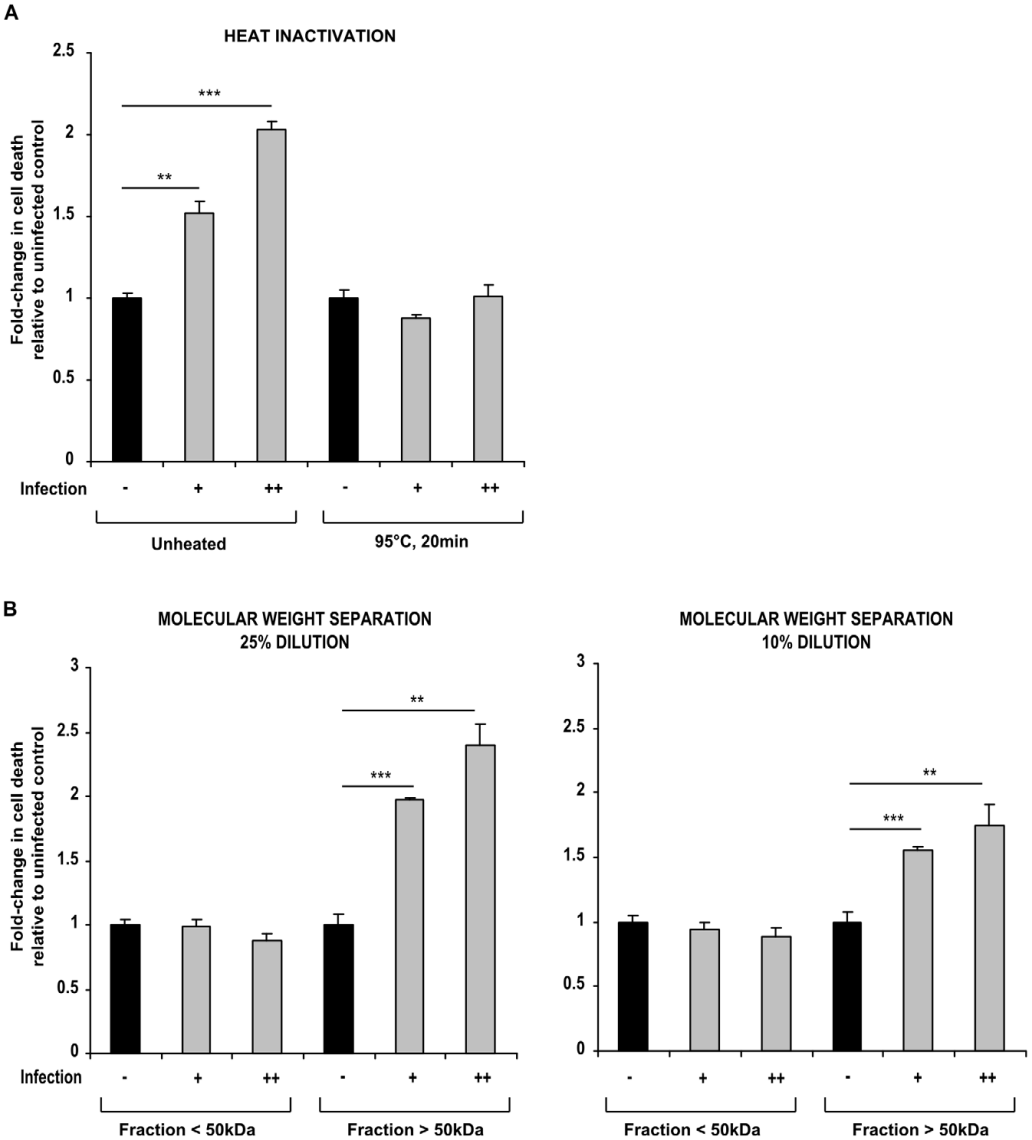
T cell death in *M.tuberculosis*-infected macrophage culture supernatants is mediated by heat-sensitive factor(s) greater than 50 kDa in molecular size. (A) Heat inactivation (95°C, 20 min) of supernatants from low MOI and high MOI-infected macrophages (shaded bars, infection + and ++ respectively) inhibited induction of Jurkat T cell death. (B) Death-inducing factor(s) in infected macrophage supernatants were retained in the >50 kDa fractions following molecular size separation, and killed Jurkat T cells in a dose-dependent manner (25% and 10% dilution). Data shown in each panel are from one representative experiment, showing fold-change in cell death ± SEM in low MOI and high MOI-infected macrophage supernatants (shaded bars, infection + and ++, respectively) relative to uninfected control (closed bars), from incubations performed in triplicate. Each experiment was repeated a minimum of three times. ** =  p<0.01, *** =  p<0.001, Student’s *t* test relative to uninfected control.

### Macrophage-free *M. tuberculosis* Bacilli are not Sufficient to Signal T Cell Death

Mtb bacilli alone, and without macrophages, were insufficient to cause toxicity to T cells; in PBTLs coincubated for 24 h with live bacilli at 1∶1, 1∶5 and 1∶10 ratios; 12.89±0.94%, 11.72±1.55% and 12.01±0.34% cell death was observed, respectively, and none of these was significantly higher than the medium-only control, where 9.21±0.36% cell death occurred (fig. 3A). This result was also seen in Jurkats coincubated for 24 h with live bacilli at 1∶1, 1∶5 and 1∶10 ratios; 6.50±0.28%, 4.62±0.34 and 5.82±0.57% cell death was observed, and again this was not significantly higher than in cells incubated in the absence of Mtb, where 3.77±0.38% death was observed (fig. 3B). Culture medium from Mtb H37Ra growing in the log phase was also applied Jurkat T cells, in order to investigate whether factors released during normal growth of the bacilli in culture broth could perturb viability. No significant increase was observed in cells incubated in complete RPMI supplemented with 10, 25 or 50% of Mtb-conditioned medium; 1.93±0.12%, 2.05±0.07% and 4.37±0.88% cell death was observed, respectively, whilst 2.86±0.22% was observed in the unsupplemented control. 1 µM staurosporine induced significantly (p<0.001) enhanced cell death, at 58.53±0.18% (fig. 3C).

### T Cell Death Mediated by Secreted Antigens of *M. tuberculosis*

To examine whether T cell death in our system was attributable to other specific secreted proteins from Mtb, and since such secreted factors have previously been shown to alter immune cell survival [25], [26], [27] and activity [28], we exposed Jurkat cells to a panel of Mtb-derived factors. In Jurkat cells incubated in the presence of ESAT-6 at concentrations of 10 µg/ml, 1 µg/ml and 0.1 µg/ml, 58.33±0.58%, 12.43±0.55% and 7.04±0.23% cell death was observed. At 10 µg/ml and 1 µg/ml, cell death was significantly (P<0.001) elevated above that of the vehicle control, where 6.35±0.16% death was observed. Staurosporine 1 µM was used as a positive cell killing stimulus, and induced 64.63±1.34% death. Elevated death was not observed in T cells incubated with other secreted antigens such as 16 kDa, 45 kDa, Antigen 85 complex, GroES, ManLAM or Phos1 (fig. 4A–G). To examine whether ESAT-6 could be the principal mediator of T cell death in our system, we performed AM infections using Mtb H37Ra in parallel with *M.bovis* BCG, then cultured Jurkat T cells in the resulting cell-free supernatants as described previously. In uninfected AM supernatants, T cell death was 9.56±0.22%; this significantly increased to 14.39±1.38% (p<0.05) and 18.30±0.31% (p<0.001) in low and high MOI H37Ra-infected AM supernatants, respectively. In low and high MOI BCG-infected AM supernatants, T cell death also increased significantly (p<0.01) to 12.38±0.52% and 17.1±0.91%, respectively (fig. 4H). No significant difference was found when T cell death in H37Ra-infected AM supernatants at low or high MOI was compared with that in BCG-infected AM supernatants at the same MOIs.

### Cell Death Induction by Infected Macrophage Supernatants is Caspase-dependent, but not Mediated by TNF-α or Fas

To further delineate the factors responsible for T cell killing in macrophage conditioned medium, a number of blocking strategies were employed. Firstly, addition of the pan-caspase inhibitor, Z-VAD-FMK to infected macrophage culture supernatants before incubation with T cells attenuated cell death, indicating that the effect was caspase-dependent. In cells incubated without inhibitor, cell death in medium from uninfected macrophages was 13.66±0.45%, which increased significantly (p<0.001) to 24.39±0.61% and 25.07±0.81% in low MOI and high MOI supernatants respectively. Following incubation in the same supernatants after addition of 20 µM Z-VAD-FMK, cell death was 2.01±0.19%, 1.88±0.03% and 2.56±0.03% in uninfected, low MOI and high MOI supernatants, respectively (fig. 5A). In positive controls, 1 µM staurosporine induced 59.45±0.97% cell death, which was significantly (p<0.001) mitigated by coincubation with 20 µM Z-VAD-FMK, to 28.14±1.35% (fig. 5D).

Secondly, supernatants from infected macrophages were applied to T cells in the presence of TNF-α blocking antibody, at a concentration of 5 µg/ml, or isotype IgG at the same concentration. In the presence of isotype control antibody, 11.29±0.10% death occurred in cells incubated in uninfected macrophage supernatants. This increased significantly (p<0.001) to 17.79±0.38% and 20.63±0.45% in cells incubated in low and high MOI-infected macrophage supernatants. This cytotoxicity was not inhibited by coincubation with anti-hTNF-α; cell death increased significantly from 10.72±0.63% in the uninfected supernatants up to 17.13±0.13% and 19.18±0.51% (p<0.001 in both cases) in low and high MOI-infected macrophage supernatants, respectively (fig. 5B). In positive controls, 5 ng/ml recombinant human TNF-α +0.2 µg/ml cycloheximide caused 26.96±1.82% cell death in Jurkats incubated in the presence of isotype control IgG, which was significantly (p<0.01) reduced to 12.22±0.23% in the presence of anti-hTNF-α (fig. 5D).

Anti-Fas blocking antibody, or isotype control IgG were added at a concentration of 2.5 µg/ml to supernatants. Cell death increased significantly from 6.16±0.65% in uninfected macrophage supernatants up to 21.44±0.12% (p<0.001) in low MOI, and 23.03±0.38% (p<0.001) in high MOI-infected macrophage supernatants in the presence of isotype control IgG, and this pattern was recapitulated in supernatants in the presence of anti-Fas, where cell death increased from 6.55±0.42 in uninfected supernatants to 21.79±1.13% and 25.70±0.58% in low and high MOI-infected supernatants, respectively (p<0.001 in both cases) (fig. 5C). In positive controls, recombinant human Fas ligand induced 51.48±0.43% cell death, which was significantly reduced to 34.84±0.88% (p<0.001) (fig. 5D).

### The Cell Death Signal of Infected Macrophage Supernatants is Heat-labile and Mediated by Factor(s) Greater than 50 KiloDaltons in Molecular Size

To investigate the composition of the signalling factor(s) that induced T cell death, supernatants from uninfected and infected macrophages were heat-denatured at 95°C for 20 min, and then applied to Jurkat T cells. Following 24 h incubation, cell death was measured by Hoechst/PI staining, and expressed as fold-change in death relative to uninfected control. Unheated supernatants from low and high MOI-infected macrophages caused a significant (p<0.01, p<0.001 respectively) fold-increase in cell death to 1.52±0.07 and 2.04±0.05 of that in the uninfected control supernatants. This effect was mitigated by heat denaturing; in Jurkats incubated in heat-treated supernatants, fold-change in cell death was 0.88±0.02 and 1.01±0.08 of that in uninfected control supernatants (fig. 6A).

Subsequently, culture supernatants were passed through 50 kDa molecular weight cutoff filters, diluted in fresh medium as outlined in methods, and then used to culture Jurkat T cells. Cell death was measured by PI/Hoechst staining and expressed as fold-change relative to the uninfected supernatants. Following 24 h incubation, death was not increased by the filtrate containing molecules below 50 kDa in size, where low and high MOI supernatant <50 kDa fractions at 25% dilution did not induce any significant fold-change in death compared to that of the control uninfected supernatant (0.99±0.05 and 0.87±0.06 fold-change relative to control, respectively). Conversely, a significant (p<0.001 low MOI, p<0.01 high MOI) increase was observed in death in cells incubated in the low and high MOI >50 kDa fractions at 25% dilution, causing a change of 1.97±0.02-fold, and 2.40±0.17-fold respectively, relative to control (fig. 6B). These results were recapitulated at 10% dilution of the supernatant filtrates and retentates; again no significant increase in cell death was observed in T cells cultured in the <50 kDa fractions of low and high MOI supernatants (0.99±0.05 and 0.87±0.06 fold-change relative to uninfected control), whilst cell death induction was maintained in the >50 kDa fractions; low MOI supernatant retentate caused a 1.56±0.04-fold increase in cell death, whilst high MOI supernatant retentate caused a 1.75±0.08-fold increase, both of which were significantly (p<0.001 and p<0.01 respectively) higher than control (fig. 6B).

## Discussion

In our tuberculosis patients, we found that lymphopenia was prevalent and reversible after treatment for this infectious disease. This systemic lack of T cell function may be linked to the poor performance of clinical tests for Mtb infection in tuberculosis patients that rely on lymphocyte function - such as the Tuberculin skin test and the Interferon-γ release assay. To study the effect of Mtb-infected macrophages on lymphocyte depletion, we examined T cell viability when exposed to the cell-free culture supernatant of infected phagocytes. Our results show that culture supernatants from Mtb-infected human macrophages induce contact-independent T cell apoptosis, in a dose-dependent manner. We found that T cell survival was unaffected by (macrophage-free) Mtb bacilli or their secreted products in bacterial culture, with the exception of ESAT-6, which reliably induced T cell death. However, subsequent experiments showed networked T cell death occurred in our system even when culture supernatants of AMs infected with the ESAT-6-deficient *M.bovis* BCG were used. Although T cell death in macrophage supernatants was caspase-dependent, it was not mediated by TNF-α or Fas. Further characterisation of the death-inducing factor(s) revealed them to be heat-labile and greater than 50 kDa in molecular size.

During infection by *M.tuberculosis*, several strategies facilitate pathogenesis and avoidance of bacillary destruction, including perturbation of phagosomal maturation [23], manipulation of phagocyte viability in order to preserve the replicative environment and subsequently facilitate release of newly-produced bacilli [29] and downmodulation of responsiveness to activating signals [30]. Alteration of host cell signalling responses can also elicit favourable conditions for Mtb [31], and secreted virulence factors have also been shown to play a role in modulating immune cell signalling [32]. Inducing death of host T cells, thereby disrupting their interaction with infected phagocytes, may be a further mechanism by which Mtb evades host immunity.

Elimination of immune effector cells in the proximity of infected phagocytes is thematically consistent with the concept of establishing the Mtb infection site as an immune-privileged location [33], [34], and it is clear that multiple mechanisms contribute to this process. Contact-dependent death of nearby, non-infected cells has been shown in previous studies [14], [16], [18], but in our system an additional mode of T cell killing was found, which was mediated by soluble factors. Other distinct networked cell signalling effects following Mtb infection are also known to occur - for example, Green *et al.* demonstrated that conditioned medium from infected monocytes elicited release of matrix metalloproteinase from microglia, representing a novel mechanism of pathology arising from TB disease in the central nervous system [35].

Since T cells arriving at the site of infection are likely to be activated, further investigation is now warranted into the importance of activation status on Mtb infection- induced cell death. However, whilst Sharma *et al*. have shown that activated T cells undergo increased apoptosis when exposed to Mtb-infected macrophages [16], our own experiments exposing unstimulated primary T cells to supernatants from infected macrophages have also shown increased cell death. It is therefore still unclear to what degree activation status influences infection-induced networked cell death, and further examination of the susceptibility of particular subsets of T cells, such as CD4+ or CD8+, would also be of utility.

Exposure to viable Mtb bacilli did not cause an increase in T cell death during our coculture experiments, nor did incubation in their spent culture medium. However, changing physiological conditions, such as those encountered by Mtb upon phagocytosis for example, may lead to the expression of a distinct subset of genes that mediate bacillary survival and persistence [36], [37]. Our model has excluded a role for Mtb bacilli in isolation causing T cell killing, but virulence factors that are upregulated following intracellular infection of phagocytes might still contribute to networked T cell death. We saw that ESAT-6 was sufficient to cause increased T cell death. However, as mentioned above, it is unlikely to be the major contributor to death in our system, since experiments demonstrated the apoptosis-inducing effects of supernatants from BCG-infected macrophages. BCG does not express ESAT-6 due to deletion of the RD1 locus [38]. In addition, our molecular weight separation experiments show that the proapoptotic signals are mediated by infected macrophage supernatant factors greater than 50 kDa in size. Although ESAT-6 may be present in >50 kDa complexes with other Mtb virulence factors [39], [40], it is still unlikely to be the death factor in our presented experiments using Mtb H37Ra. This is because H37Ra does not secrete abundant ESAT-6 due to a S219L point mutation in the predicted DNA binding region of the regulator PhoP [41].

TNF-α and Fas/Fas Ligand interactions have both been shown to regulate cell death during mycobacterial infections [17]. Key studies by Sharma *et al*., and Hirsch *et al*., had previously implicated TNF-α and Fas in deletion of T cells during Mtb infection in a contact-dependent manner [16] and in a mixed PBMC-Mtb coincubation system [15], respectively. The latter implicated TNF-α, TGF-β, Fas and modulation of Bcl-2 expression in mediating changes in T cell viability in Mtb infection, but also showed that the mechanisms varied between patients. When specifically interrogating the effects of soluble mediators in our own supernatant transfer model, we found that the addition of blocking antibodies against either TNF-α or Fas to the infected macrophage supernatants did not reduce killing of T cells. It is therefore clear that an additional TNF-α- and Fas- independent mode of action may exist, that is contact-independent, and can cause T cells to undergo apoptosis, mediated exclusively by soluble factors released from infected phagocytes.

Heat treatment showed that the signal(s) present in infected macrophage supernatants responsible for T cell killing were heat-labile, and we subsequently employed a molecular size separation protocol in order to more clearly delineate these factor(s). In these experiments, the cytotoxic effect separated to the >50 kDa fraction, and suggested that a large protein or multimeric cytokines could be responsible. The lack of increased death in cells incubated in the <50 kDa fraction indicated that small molecule mediators such as nitric oxide [42] or ATP [43], though both relevant in control of tuberculosis infection in macrophages [7], [44], are not responsible for the T cell death observed in our experimental system. Furthermore, T cell killing activity of infected supernatants was retained even when the >50 kDa fractions were diluted to 10% in fresh medium, precluding the possibility that simple nutrient depletion within the supernatants was responsible for the cytotoxic effect.

Networked T cell death by soluble host- and/or pathogen-derived mediators released during Mtb infection may contribute to the T cell lymphopenia observed in patients with active TB disease; furthermore, premature deletion of T cells as they approach the infection site could inhibit the delivery of activating cytokines such as IFN-γ, thus rendering the infected phagocytes incompetent to degrade and clear the pathogen. A similar mode of infection-induced networked death signalling occurs during infection by other intracellular bacteria such as *Chlamydia trachomatis* [45]. In the case of *M.tuberculosis*, this phenotype likely represents one of several manipulations of host immunity that enable persistence. It is possible that by protecting T cells from this toxic influence of infected macrophages *in-vivo*, the balance may be tipped towards preserving immune effector capacity, and improved disease resolution.

## Supporting Information

Figure S1 **Automated cell death image analysis.** Images were acquired at 10× magnification using 360/460 nm and 535/620 nm excitation/emission filter sets to detect fluorescence from Hoechst and PI staining, respectively (A; bar 50 µm, Hoechst/PI intensity shown for the same field of view for Jurkat T cells incubated in control uninfected macrophage supernatant). Normal nuclei were distinguishable from condensed nuclei (example arrow 1), whilst mitotic cells did not show increased Hoechst staining, thus precluding false-positives (arrow 2). PI staining was also clearly distinguishable (arrow 3). Intensity measurements for Hoechst and PI in each cell were analysed on a cell-by-cell basis; cells with condensed nuclei were identified by an elevated coefficient of variance of nuclear intensity (nuclear intensity CV; a measure of the degree of variation above the nuclear intensities of other cells in that field) above a user-defined threshold, and PI-positivity was also identified by intensity above a user-defined threshold. Using a decision-tree protocol, cells were categorised into four classes: Live, PI-positive, condensed nucleus, and condensed nucleus + PI. The latter three categories were then summed to give percentage cell death for each field of view (B). 12 fields of view were acquired per treatment, and each treatment was performed in triplicate.
(TIF)
