# Supplementary figures and images for: Networked T Cell Death following Macrophage Infection by Mycobacterium tuberculosis
Source: PLoS One. 2012 Jun 4;7(6):e38488. doi: 10.1371/journal.pone.0038488 (PMC3366923; doi:10.1371/journal.pone.0038488)

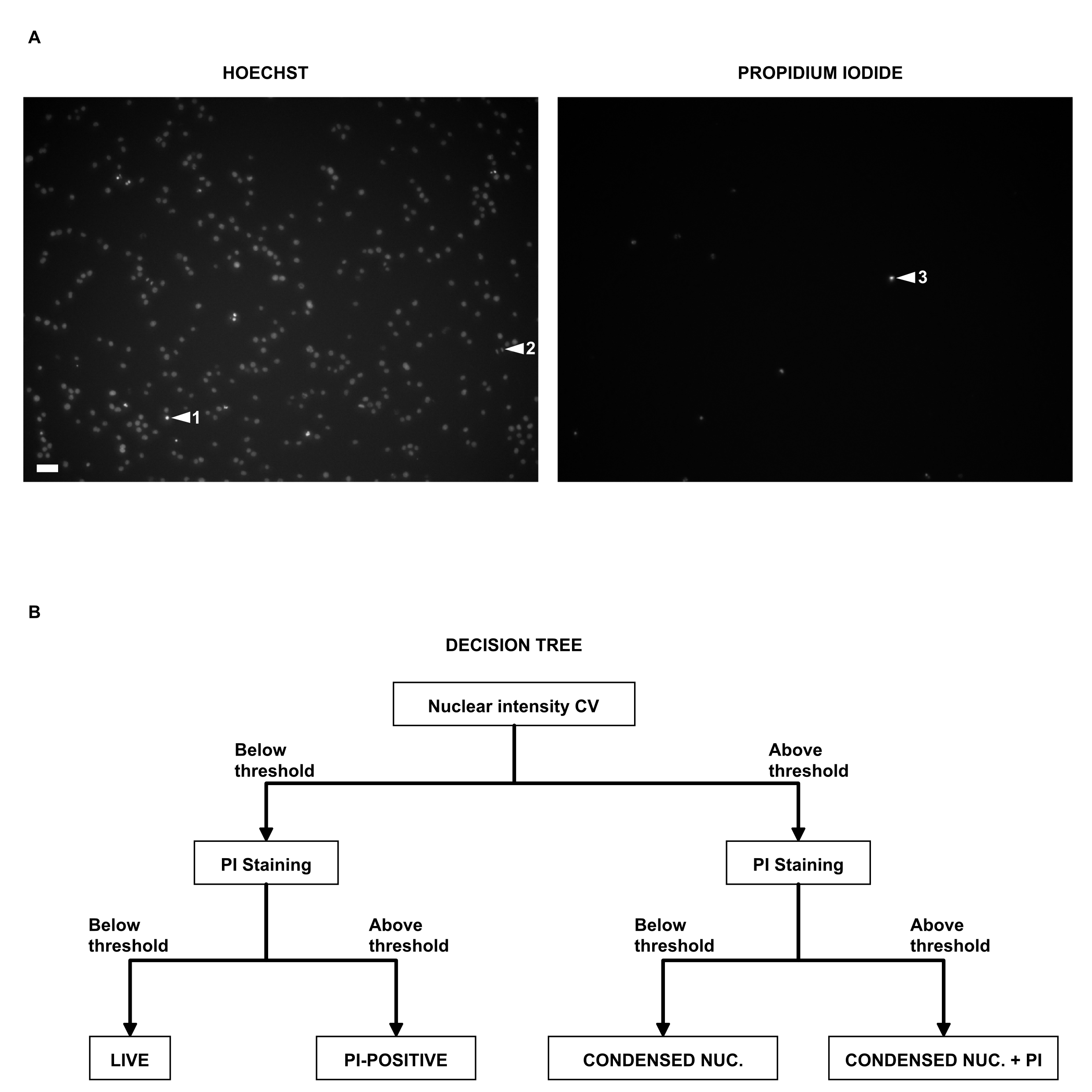

Supplement: Figure S1 — Automated cell death image analysis. Images were acquired at 10× magnification using 360/460 nm and 535/620 nm excitation/emission filter sets to detect fluorescence from Hoechst and PI staining, respectively (A; bar 50 µm, Hoechst/PI intensity shown for the same field of view for Jurkat T cells incubated in control uninfected macrophage supernatant). Normal nuclei were distinguishable from condensed nuclei (example arrow 1), whilst mitotic cells did not show increased Hoechst staining, thus precluding false-positives (arrow 2). PI staining was also clearly distinguishable (arrow 3). Intensity measurements for Hoechst and PI in each cell were analysed on a cell-by-cell basis; cells with condensed nuclei were identified by an elevated coefficient of variance of nuclear intensity (nuclear intensity CV; a measure of the degree of variation above the nuclear intensities of other cells in that field) above a user-defined threshold, and PI-positivity was also identified by intensity above a user-defined threshold. Using a decision-tree protocol, cells were categorised into four classes: Live, PI-positive, condensed nucleus, and condensed nucleus + PI. The latter three categories were then summed to give percentage cell death for each field of view (B). 12 fields of view were acquired per treatment, and each treatment was performed in triplicate. (TIF) [file pone.0038488.s001.tif]
